# Cardioprotective effects of liposomal resveratrol in diabetic rats: unveiling antioxidant and anti-inflammatory benefits

**DOI:** 10.1080/13510002.2024.2416835

**Published:** 2024-11-04

**Authors:** Ahmed Z. Alanazi, Mohammed Alqinyah, Abdullah S. Alhamed, Hanan Mohammed, Mohammad Raish, Khaldoon Aljerian, Jawza F. Alsabhan, Khalid Alhazzani

**Affiliations:** a Department of Pharmacology and Toxicology, College of Pharmacy, King Saud University, Riyadh, Saudi Arabia; b Department of Pharmaceutics, College of Pharmacy, King Saud University, Riyadh, Saudi Arabia; c Department of Pathology, College of Medicine, King Saud University, Riyadh, Saudi Arabia; d Department of Clinical Pharmacy, College of Pharmacy, King Saud University, Riyadh, Saudi Arabia

**Keywords:** Diabetic cardiomyopathy, inflammation, NF-kB, oxidative stress, apoptosis, catalase, glutathione peroxidase

## Abstract

As a consequence of chronic hyperglycemia, diabetes complications and tissue damage are exacerbated. There is evidence that natural phytochemicals, including resveratrol, a bioactive polyphenol, may be able to reduce oxidative stress and improve insulin sensitivity. However, resveratrol's limited bioavailability hampers its therapeutic effectiveness. By using liposomes, resveratrol may be better delivered into the body and be more bioavailable. The objective of this study was to assess the cardioprotective potential of liposome-encapsulated resveratrol (LR) in a streptozotocin-induced (STZ) diabetic rat model. Adult male Wistar rats were categorized into five groups: control, diabetic, resveratrol-treated (40 mg/kg), liposomal resveratrol (LR)-treated (20 mg/kg) and liposomal resveratrol (LR)-treated (40 mg/kg) for a five-week study period. We compared the effects of LR to those of resveratrol (40 mg/kg) on various parameters, including serum levels of cardiac markers, tissue levels of pro-inflammatory cytokines, nuclear transcription factor, oxidative stress markers, and apoptotic markers. LR treatment in STZ-diabetic rats resulted in notable physiological improvements, including blood glucose regulation, inflammation reduction, oxidative stress mitigation, and apoptosis inhibition. LR effectively lowered oxidative stress and enhanced cardiovascular function. It also demonstrated a remarkable ability to suppress NF-kB-mediated inflammation by inhibiting the pro-inflammatory cytokines TNF-α and IL-6. Additionally, LR restored the antioxidant enzymes, catalase and glutathione peroxidase, thereby effectively counteracting oxidative stress. Notably, LR modulated apoptotic regulators, including caspase, Bcl2, and Bax, suggesting a role in regulating programmed cell death. These biochemical alterations were consistent with improved structural integrity of cardiac tissue as revealed by histological examination. In comparison, resveratrol exhibited lower efficacy at an equivalent dosage. Liposomal resveratrol shows promise in alleviating hyperglycemia-induced cardiac damage in diabetes. Further research is warranted to explore its potential as a therapeutic agent for diabetic cardiovascular complications and possible cardioprotective effects.

## GRAPHICAL ABSTRACT

**Figure:**
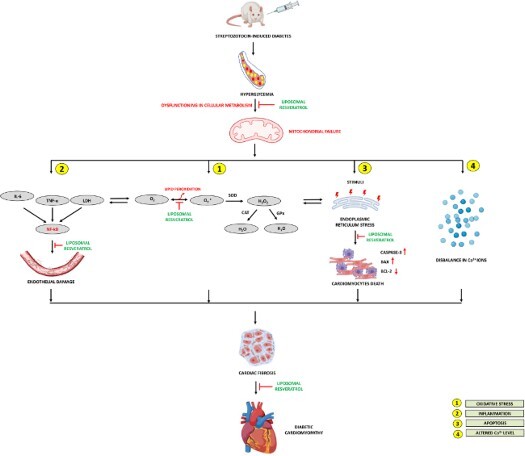

## 1. Introduction

The global burden of diabetes has escalated significantly over recent decades, paralleling the rise in obesity and sedentary lifestyles. The International Diabetes Federation estimated a worldwide diabetes prevalence of 463 million in 2019, projected to reach 700 million by 2045 [1]. Such rapid upsurge has been described as a modern-day epidemic attributed to population growth, aging, urbanization, and associated lifestyle changes. Developing nations are disproportionately affected by the diabetes epidemic, with China, India, and the USA accounting for the highest number of cases [2]. However, incidence continues to increase across high as well as low-income regions. The substantial disease burden and healthcare costs associated with diabetes make it a pressing global public health issue [3].

The persistent elevation of blood glucose levels in diabetes triggers a cascade of detrimental effects on the heart known as Diabetic Cardiomyopathy (DCM) [4]. It stands as a distinct cardiac complication of diabetes mellitus, characterized by progressive cardiac dysfunction in the absence of other underlying heart diseases. The pathophysiological underpinnings of DCM lie in a complex interplay between hyperglycemia-induced oxidative stress, inflammation, and apoptosis, ultimately leading to cardiomyocyte demise and subsequent cardiac remodeling [4–6]. Chronic hyperglycemia triggers a cascade of events, initiating the production of reactive oxygen species (ROS) through multiple pathways, including the generation of advanced glycation end products (AGEs), activation of the polyol and hexosamine pathways, and dysregulation of protein kinase C (PKC) signaling [5]. This accumulation of ROS exacerbates mitochondrial dysfunction, culminating in cardiomyocyte death and the initiation of cardiac remodeling processes, namely hypertrophy and fibrosis [4].

Current diabetes management continues to emphasize lifestyle measures like dietary improvements, weight reduction, smoking cessation, and regular physical activity. Multiple pharmaceutical options are prescribed for regulating blood sugar levels. However, maintaining consistent glycemic control remains difficult, and existing treatments often lose effectiveness over time. Furthermore, existing therapies often lose effectiveness over time, and current interventions fall short in preventing or adequately slowing the progression of secondary diabetic complications affecting the vasculature, kidneys, nerves, and retina.

Consequently, research continues to focus on development of novel antidiabetic agents, particularly from natural sources, with efficacy and safety profiles superior to existing drugs. Resveratrol is a polyphenolic stilbene compound found in various dietary sources including grapes, berries, peanuts, and red wine [7]. Extensive research over the past two decades has uncovered an array of therapeutic effects and molecular targets of resveratrol. It exhibits potent antioxidant properties by scavenging reactive oxygen and nitrogen species directly and enhancing endogenous antioxidant defenses. Resveratrol activates important transcription factors related to antioxidant gene expression such as nuclear factor erythroid 2 – related factor 2 (Nrf2) [8]. It reduces vascular oxidative stress and inhibits LDL oxidation to prevent atherosclerosis [9]. Through diverse mechanisms, resveratrol protects against DNA damage, mutagenesis, and cancers. It mimics certain impacts of calorie restriction by activating sirtuins and energy sensors like AMPK [10]. Resveratrol, known for its multifaceted effects, not only suppresses cancer cell proliferation and metastatic potential through various pathways but also demonstrates neuroprotective, hepatoprotective, renal-protective, and anti-aging properties [11].

Of particular relevance, resveratrol has demonstrated anti-hyperglycemic [12], anti-inflammatory [13], and anti-apoptotic properties in preclinical models that support its potential as a therapeutic agent for diabetes [14]. Resveratrol enhances insulin sensitivity, increases glucose uptake, improves insulin signaling, preserves beta cell function, and suppresses hepatic gluconeogenesis [15]. Resveratrol reduces inflammatory cytokines, inhibits NF-κB activation, and ameliorates diabetic renal, retinal, neural, and vascular injury [16]. It also attenuates diabetes-associated activation of apoptotic pathways. Integrated effects of resveratrol on pathways related to glycemic control, inflammation, oxidative stress, and cell survival underlie its organ-protective benefits against diabetes and associated complications [17]. Bioavailability studies show oral resveratrol undergoes extensive first-pass metabolism resulting in low systemic levels. Novel formulations like nanoparticles and liposomes are being developed to enhance its bioavailability [18].

Combining resveratrol with other antidiabetic agents also offers synergistic possibilities. Elucidating resveratrol’s influence on these critical pathological parameters and processes could enhance understanding of its cardioprotective effects against diabetes-associated complications. This study aimed to assess the cardioprotective potential of liposome-encapsulated resveratrol (LR) in a streptozotocin-induced (STZ) diabetic rat model for studying the effects of liposomal resveratrol complex in diabetic rats to improve the understanding of their antioxidant and anti-inflammatory mechanisms. This preclinical study was therefore undertaken to systematically elucidate the antidiabetic and biomarker-modulating effects of resveratrol supplementation in an experimental rat model of streptozotocin (STZ)-induced diabetes. We evaluated the impact of daily oral liposomal resveratrol administration on a panel of interlinked diabetic biomarkers related to glycemic control, dyslipidemia, inflammation, oxidative stress, and apoptosis.

## 2. Materials and methods

### 2.1. Characterization of particle size, polydispersity index (PDI), and entrapment efficiency

LR was diluted 100 times in pre-filtered and double-distilled water for this experiment. A Malvern Zetasizer NanoZS device operating at 25 °C was used to measure the particle size and PDI of liposomes loaded with formulated Resveratrol [19]. LR was evaluated by centrifugation of samples for 3 hour at 15,000 rpm [20]. The supernatant from the samples was pipetted out and examined for drug content by UV spectrophotometer at 322 nm [21]. The below equation was used to calculate EE [22].

$$\mathrm{EE}\text{\%}=\frac{\left(\mathrm{Total}\;\mathrm{RES}-\mathrm{RES}\;\mathrm{detected}\;\mathrm{in}\;\mathrm{suppernatent}\;\right)}{\mathrm{Total}\;\mathrm{RES}}x\;100$$

### 2.2. The morphological evaluation of marketed liposome-encapsulated resveratrol

The vesicle surface morphology of marketed LR was evaluated by Transmission electron microscopy (TEM). The sample was 100 times diluted and analyzed by placing a drop of the sample on a copper grid, applying phosphotungstic acid (2%) to the sample, and air-drying it. After that, the samples were viewed using TEM operated at an accelerated voltage of 80 kV [23].

### 2.3. Chemicals and materials

Streptozotocin, Resveratrol, and Liposomal Resveratrol was purchased from Sigma (St. Louis, IMO, USA) which is marketed as liposomal Trans RES® (having a particle size = 200 nm) and was purchased from Lipolife® (Drakes Lane, Chelmsford, UK). All other chemicals, ELISA kits and reagents were of the highest analytical grade commercially available.

### 2.4. Procuring animals

Thirty male Wistar rats, weighing between 250–270 g, were obtained from the Experimental Animal Care Center at the College of Pharmacy, King Saud University. These rats were housed under carefully controlled environmental conditions, which included maintaining a temperature of 22 ± 1°C, humidity levels at 50–55%, and a 12-hour light/12-hour dark cycle. They were subjected to a 7-day acclimatization period under standard laboratory conditions prior to experimentation. The rats were given unrestricted access to standard commercial rodent chow and water for the entire duration of the experiment. All experimental protocols involving animal use and handling were executed in strict accordance with the ethical regulations mentioned by the NIH’s Institute for Laboratory Animal Research guidelines for the care and use of laboratory animals (NIH Publication No. 80–23; 1996). Ethical approval for this study (KSU-SE-23-36) was duly obtained from the Institutional Animal Care & Use Committee (IACUC) at King Saud University, Riyadh, Kingdom of Saudi Arabia.

### 2.5. Induction of diabetes and experimental grouping

**Figure 1.**
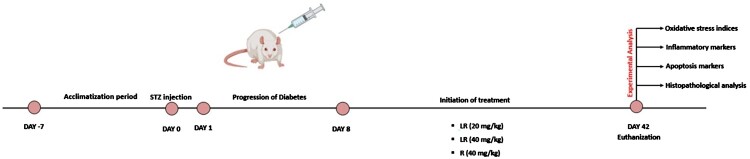
Illustration of the experimental design and diabetes induction process in the study.

Streptozotocin, a widely recognized methyl nitrosourea compound used to induce experimental diabetes mellitus in rodent models, exerts its cytotoxic effects selectively on insulin-producing pancreatic beta cells [24,25]. To induce diabetes, a single dose of streptozotocin (STZ) was administered at a dosage of 65 mg/kg to all rat groups except the control. STZ was dissolved in a 0.1 M citrate buffer solution with a pH of 4.5 to enhance its stability and bioavailability (Figure 1). The control group, in contrast, received only the citrate buffer. Two days post-STZ injection, diabetes was confirmed by measuring the fasting blood glucose levels of the rats using an Accu-Check Compact-Plus glucometer device (Roche Diagnostics, France). Rats with blood sugar levels exceeding 13.9 mmol/L (250–300 mg/dL) were included in the study. Throughout the study, the blood glucose levels of all enrolled rats were closely monitored, and all rats consistently maintained diabetic levels as per the induction criteria. Therefore, no rats were excluded based on the reversal of diabetes.

Following the confirmation of diabetes, the selected rat population was distributed into five experimental groups (n = 6 per group) as follows:

- Group 1: Control rats with no STZ injection and administered with vehicle-only
- Group 2: Diabetic rats administered with vehicle only
- Group 3: Diabetic rats administered with 20 mg/kg/day oral LR for 5 weeks starting on day 8
- Group 4: Diabetic rats administered with 40 mg/kg/day oral LR for 5 weeks starting on day 8
- Group 5: Diabetic rats administered with 40 mg/kg/day oral resveratrol for 5 weeks starting on day 8

One week after STZ administration, the diabetic rats began receiving daily oral LR/ resveratrol/ vehicle through gavage for a period of 5 weeks. Both control and STZ-treated groups received an equivalent volume of carboxymethylcellulose (CMC) solution as the vehicle.

On day 42, rats were anaesthetised and euthanized with intraperitoneal injection of ketamine HCl (92 mg/kg, Hikma Pharmaceuticals, Amman, Jordan) and xylazine (10 mg/kg, Bayer, Turkey). Blood was obtained through cardiac puncture and the samples were centrifuged at 2000 × g for 10 minutes to isolate serum. The latter was divided into aliquots and stored at −20°C for subsequent analyses. The hearts were excised and a transverse section of the extracted heart was fixed in 10% phosphate-buffered formalin (pH 7.4) for histopathological examinations. The residual cardiac tissue was briefly immersed in liquid nitrogen and stored at – 80°C for future analyses.

### 2.6. Measurement of serum biochemical markers

A panel of cardiac function biomarkers, including total protein, calcium, and LDH was measured in serum using commercially available diagnostic kits from Human, Wiesbaden, Germany. Using this kit, we evaluate the effect of LR at low and high doses, compared to resveratrol alone.

#### 2.6.1. Assessment of inflammatory mediators, transcription factor, and oxidative stress markers

Diabetes causes increased expression of transcription factors, oxidative stress, and inflammation mediators. Therefore, to evaluate the levels of inflammatory mediators, transcription factors, and oxidative stress indices in the heart, the following procedures were employed. Heart tissue was homogenized at a 10% w/v ratio in cold phosphate-buffered saline (PBS) containing proteinase inhibitors. The resulting homogenate underwent centrifugation, yielding a clear supernatant. The protein concentration within the supernatant was determined using the Bradford reagent. Equal amounts of total protein from each sample were then used to quantify the levels of NF-kB, TNF-alpha, and IL-6 utilizing ELISA kits sourced from R&D Systems Inc., Minneapolis, MN, USA, following the guidelines provided by the manufacturer. This normalization to protein levels ensures the reliability and comparability of the biochemical data across different experimental groups.

#### 2.6.2. Assessment of antioxidant activities

Malondialdehyde (MDA) is a marker of lipid peroxidation, a process that results in the formation of reactive aldehydes due to oxidative damage to unsaturated fatty acids. Therefore, to determine the levels of TBARS (Thiobarbituric Acid Reactive Substances), we utilized diagnostic kits procured from Cayman Chemical Co., USA, and followed the protocols provided by the manufacturer. By determining whether LR is successful in restoring MDA levels under diabetic conditions, this assay enables us to evaluate lipid peroxidation, which provides important insight into the levels of oxidative stress.

In addition to TBARS measurements, analysis of other antioxidant enzymes was also necessary to determine their levels in diabetic conditions. To accomplish this, we collected heart post-mitochondrial supernatants for the estimation of Catalase (CAT) and Glutathione peroxidase (GPx) enzymatic activities. To perform these assessments, we employed assay kits obtained from R&D Systems Inc., USA. CAT and GPx play pivotal roles in the defense against oxidative damage by scavenging reactive oxygen species, and their activities provide essential information about the antioxidant defense mechanisms within the heart tissue. The combined assessment of these parameters offers a comprehensive evaluation of the oxidative stress burden and antioxidant defense mechanisms in diabetic cardiomyopathy.

### 2.7. Real-time PCR

For the assessment of mRNA expression in the heart, we employed the High-Capacity cDNA Archive Kit by Applied Biosystems, (located in Waltham, MA, USA). The reverse transcription of RNA into cDNA was executed following well-established protocols.

The gene expression analysis was conducted using the ABI PRISM 7500 Sequence Detection System, (Applied Biosystems). To facilitate real-time PCR analysis of particular genes, we utilized specific primers for Caspase 3, BAX, and BCL-2, with the sequences listed in Table 1. These primers were sourced from GenScript in Piscataway, NJ, USA.

**Table 1.** Sequences of primers for apoptosis-related genes.

Gene	Sequence	Accession
BAX	5’-GTTTCA TCC AGG ATC GAG CAG-3’	NG_242629
	5’-CATCTT CTT CCA GAT GGT GA-3’	
BCL-2	5’-ATCGCTCTGTGGATGACTGAGTAC-3’	NM_016993
	5’-AGAGACAGCCAGGAGAAATCAAAC-3’	
Caspase 3	5’-TTC ATT ATT CAG GCC TGC CGA GG-3’	XM_039094205
	5’-TTC TGA CAG GCC ATG TCA TCC TCA-3’	

### 2.8. Histopathological analysis

Heart tissue was processed by sectioning, embedding in paraffin wax, and slicing into 5 μm thick sections with the aid of a Leica CM3050S research cryostat, manufactured by Leica Bio-Systems in the USA. To examine histological changes, the heart sections were stained with Hematoxylin and Eosin (H&E), and subsequently examined using light microscopy. A histopathologist, who was unaware of the experimental groups, conducted an assessment of histological alterations, making comparisons between the treated and diseased groups. Multiple non-overlapping fields were examined per sample slide to assess any potential histopathological alterations induced by the experimental conditions.

### 2.9. Statistical analysis

The results are presented as the mean ± standard error of the mean (SEM). Prior to conducting parametric analyses, the normality of all datasets was assessed using the Kolmogorov–Smirnov test with an alpha level of 0.05. The normality test results indicated that the datasets were suitable for parametric analysis. Subsequently, one-way analysis of variance (ANOVA) was performed, followed by the Dunnett’s test multiple comparison *post hoc* test, to evaluate statistically significant differences between study groups. These analyses were conducted using GraphPad Prism version 8. Statistical significance was determined at a threshold of *p* < 0.05, with levels of significance denoted as *p < 0.05, **p < 0.01, and ***p < 0.001.

## 3. Results

### 3.1. Characterization and morphological evaluation of LR

**Figure 2.**
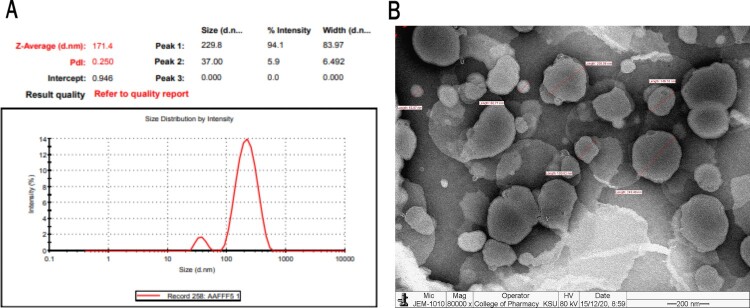
Characterization and Morphology of LR. (A) Characterization of particle size, polydispersity index (PDI), and entrapment efficiency of LR, showing a mean vesicle diameter of 159.33 ± 6.64 nm with a narrow PDI (< 0.250). (B) Transmission electron microscopy (TEM) image of LR particles, illustrating their spherical morphology and size range from 53.87–249.40 nm.

The characterization of LR revealed that the average vesicle size was 159.33 ± 6.64 nm, as shown in Figure 2A. The liposomes exhibited a narrow polydispersity index (PDI < 0.250) and a negative zeta potential of −3.94 ± 0.23. The encapsulation efficiency (%EE) was notably high, exceeding 65.83 ± 2.97%, indicating effective loading of resveratrol within the liposomes.

Morphological evaluation using transmission electron microscopy (TEM) demonstrated that the LR particles maintained a non-agglomerated shape (Figure 2B). The surface appeared smooth and clean, with liposome sizes ranging from 53.87–249.40 nm. This size distribution is particularly relevant, as particles within the range of 100–200 nm are optimal for enhancing cellular uptake via endocytosis, thereby improving the bioavailability of the encapsulated resveratrol. Figure 2B illustrates the spherical shape of the LR particles, which were consistent in size with no discernible differences.

### 3.2. Total protein, calcium and LDH levels in response to LR treatment

**Figure 3.**
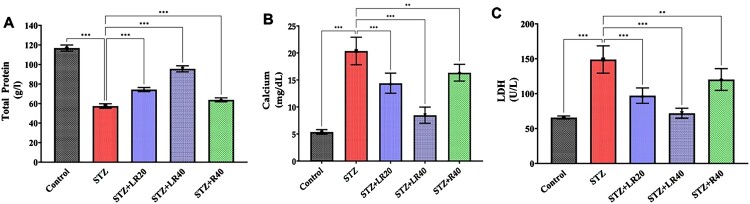
Effect of LR (20 and 40 mg/kg) and resveratrol (40 mg/kg) treatment on the serum level of (A) Protein, (B) Calcium, and (C) LDH on diabetic rats (n = 6). The data is displayed as Mean ± standard error, and significant differences are indicated as ***p < 0.001 vs. control, ***p < 0.001 vs. STZ group.

The results pertaining to serum biochemical markers are detailed in Figure 3A. The diabetic rats exhibited a significant reduction in total protein levels (*p* < 0.001) in comparison to the control group. After administering LR to animals at doses of 20 and 40 mg/kg, a remarkable elevation in protein levels was observed (*p* < 0.001) in a dose-dependent manner. However, it's worth noting that the differences between the lower dosage of LR and resveratrol alone did not reach statistical significance (*p* > 0.05). These findings suggest that LR exhibits promising potential in mitigating decreased total protein levels in conditions induced by STZ in diabetic rats.

After analyzing the impact of LR on total protein levels, it was essential to evaluate its effect on calcium homeostasis because calcium plays a vital role in cardiovascular function, and its imbalance can contribute to cardiovascular complications in diabetes. Altered calcium handling can affect vascular smooth muscle tone, blood pressure regulation, and cardiac contractility. In our study, diabetic rats treated with the vehicle showed a marked elevation in calcium levels compared to the control group (*p* < 0.001). However, LR administration at dosages of 20 and 40 mg/kg significantly reduced calcium levels (*p* < 0.001), bringing them closer to the levels observed in the control group (Figure 3B). Conversely, treatment with resveratrol alone at a dosage of 40 mg/kg resulted in a milder reduction in calcium levels (*p* < 0.01). These findings suggest that LR holds promise in reversing the elevated calcium levels in STZ-induced diabetic conditions in rats.

Additionally, we assessed lactate dehydrogenase (LDH) levels to determine the therapeutic efficacy of LR. The serum LDH levels were significantly elevated in diabetic rats compared to the control group (*p* < 0.001), indicating a marked increase in tissue damage and cellular stress associated with diabetes (Figure 3C). However, treatment with LR, particularly at a high dose, demonstrated a significant reduction in LDH levels in diabetic rats (*p* < 0.001). The low-dose resveratrol treatment (20 mg/kg) also showed a trend towards reducing LDH levels in diabetic rats, although the difference between two dosages did not reach statistical significance (*p* > 0.05). Nevertheless, the overall pattern suggests a dose-dependent effect of LR in mitigating diabetes-associated cardiac complications.

### 3.3. LR attenuates heart inflammation in diabetic rats

Considering the critical role of NF-κB in orchestrating the inflammatory response through the upregulation of pro-inflammatory genes, we sought to elucidate the intricate interplay between TNF-α, IL-6, and NF-κB in the context of diabetes-associated inflammation.

**Figure 4.**
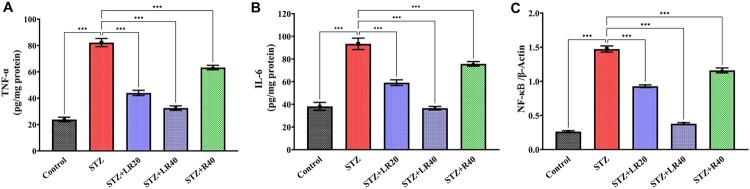
The level of (A) TNF-α (B) IL-6 (C) NF-kB was measured in the heart tissue, and concentration was expressed as pg/mg protein. The data is displayed as Mean ± standard error, and significant differences are indicated as ***p < 0.001 vs. control, ***p < 0.001, **p < 0.01 vs. STZ group.

As shown in Figure 4A-C, in the context of diabetes cardiomyopathy, our analysis unveiled a substantial upregulation in the expression of TNF-alpha (*p* < 0.001), IL-6 (*p* < 0.001), and NF-κB (*p* < 0.001) within the heart tissues of the vehicle-treated rats compared to their respective control groups. Upon introducing LR dosages of 20 and 40 mg/kg to the diabetic groups, there was a notable dose-dependent reduction in the levels of these inflammatory markers (*p* < 0.001) in comparison to the STZ-group. However, the decrease in these inflammatory markers was less pronounced when resveratrol was administered alone at a dosage of 40 mg/kg. The findings suggest that liposomal resveratrol may possess superior anti-inflammatory properties in diabetic cardiomyopathy compared to resveratrol alone at an equivalent dosage. Moreover, a dose-dependent relationship was observed, with higher concentrations of LR demonstrating more pronounced beneficial effects.

### 3.4. Influence of LR treatment on markers of oxidative stress

**Figure 5.**
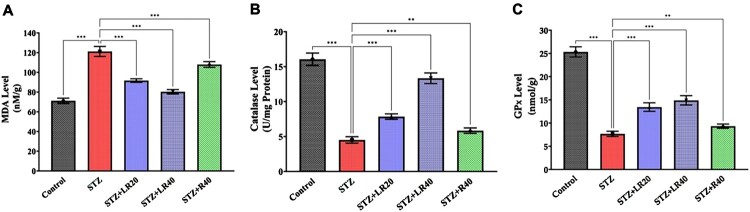
Comparative analysis of (A) MDA (B) Catalase, and (C) GPx levels in STZ-induced diabetic rats post liposomal resveratrol and resveratrol treatment at different concentrations. The data is displayed as Mean ± standard error, and significant differences are indicated as ***p < 0.001 vs. control, ***p < 0.001, **p < 0.01 vs. STZ group.

Figure 5A illustrates the impact of diabetes on lipid peroxidation. MDA levels, a marker of oxidative stress, were significantly elevated in the STZ (diabetic) group compared to the control group (*p* < 0.0001), indicating increased oxidative damage in diabetic rats. However, treatment with LR20 and LR40 effectively reduced MDA levels compared to the STZ group (*p* < 0.001), suggesting a protective effect against oxidative stress. Notably, the inhibitory effect on MDA formation was comparable for both LR doses (*p* > 0.05).

In our study, we also assessed the effects of diabetes and resveratrol treatment on antioxidant enzyme levels. Catalase and Glutathione Peroxidase are crucial antioxidant enzymes that protect cells from free radicals-induced oxidative damage. The level of both enzymes was significantly lower in the STZ group compared to the control group (*p* < 0.001), indicating impaired antioxidant defense in diabetic rats. On the other hand, the administration of LR at doses of 20 and 40 mg/kg led to a notable elevation (*p* < 0.001) in enzyme activity following a dose-dependent pattern. In contrast, the sole administration of resveratrol also caused comparable restoration (*p* < 0.01) of enzyme activity in diabetic rats compared to the STZ-group. These findings suggest that LR, particularly when administered at the increased dosage, shows promising potential in mitigating oxidative stress biomarkers in conditions induced by STZ in diabetic rats (Figure 5 A-C).

### 3.5. Inhibition of apoptosis in diabetic rats treated with LR

**Figure 6.**
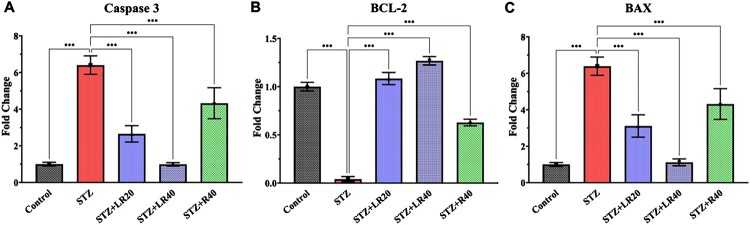
The level of (A) Caspase 3 (B) Bcl2 (C) Bax as measured in the heart homogenate through real-time PCR. The data is displayed as Mean ± standard error, and significant differences are indicated as ***p < 0.001 vs. control, ***p < 0.001 vs. STZ group.

To evaluate the potential influence of apoptotic markers on the protective effects of liposomal resveratrol (LR) against oxidative damage in the hearts of diabetic animals, we conducted a real-time PCR analysis of Bax, caspase 3, and Bcl2 expression in diabetic rats (Figure 6A-C). Our results demonstrated that diabetic rats displayed increased levels of caspase 3 and Bax protein expression in their heart tissues (*p* < 0.001), while the expression of Bcl2 was significantly reduced (*p* < 0.001). These findings imply the presence of heart dysfunction in diabetic conditions. Significantly, the administration of LR at dosages of 20 and 40 mg/kg remarkably reversed this trend. It effectively reduced the expression of caspase 3 and Bax (*p* < 0.001) while restoring the expression of Bcl2 (*p* < 0.001) when compared to the untreated diabetic rats.

### 3.6. Effect of LR on histopathological alterations

**Figure 7.**
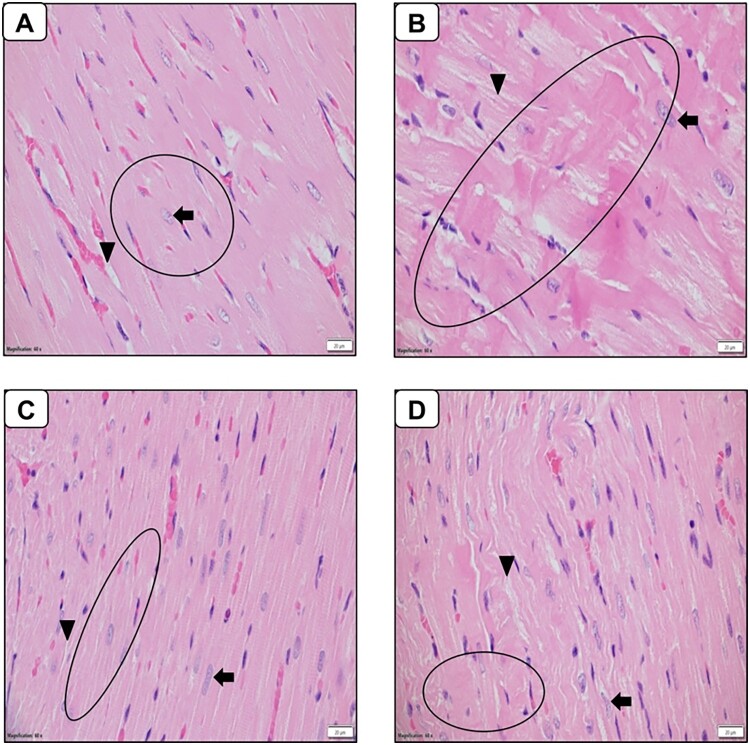
Histopathological changes in heart sections (60X). Cardiac sections from (A) control rats, (B) streptozotocin rats (C) LR-treated rats (40 mg/kg) (D) Resveratrol-treated rats (40 mg/kg) were stained with hematoxylin and eosin and assessed for the extent of histological alterations. The circles highlight myocardiocyte contraction bands, suggesting necrosis in streptozotocin-treated rats. Arrowheads point to the inter myocardiocyte space, indicating potential fibrosis in these rats. Arrows identify the nucleus of myocardiocytes, which showed irregular shapes in streptozotocin-treated rats. Treatment with LR reduced these manifestations observed in streptozotocin-treated rats. Photomicrographs are indicative of histopathology of six animals (n = 6) per experimental group.

As evidenced by the results obtained through hematoxylin and eosin (H&E) staining (Figure 7), the cardiomyocytes within the myocardium of the control group rats exhibited characteristic features of central, oval-shaped single nuclei and a regular arrangement of cardiac myofibrils. Conversely, in the STZ-induced diabetic rat group, distinct deviations were noted, including nuclei deformities, interstitial edema, and localized cytoplasmic vacuolization. Additionally, there was an irregular dispersion of cardiac myofibrils when compared to the normal control rats, collectively suggesting the presence of ventricular cardiac hypertrophy. Furthermore, there was clear evidence of substantial fibroblast infiltration in the STZ-induced diabetic group. Significantly, upon oral administration of LR at dosage of 40 mg/kg body weight, the myocardial nuclei and cardiac myofibrils’ structural integrity was notably reinstated, with a considerable reduction in fibroblast infiltration and protein degradation. These findings collectively indicate a reduction in both cardiac and ventricular hypertrophy.

## 4. Discussion

Resveratrol, a polyphenolic molecule that has grabbed emphasis over the past decade due to its antioxidant properties, has been reported to provide pharmacological protection against an array of diseases [26–28]. However, clinical investigations only demonstrated little benefit in clinical studies, which is principally due to resveratrol's bioavailability and pharmacokinetics. Because of its instability and low water solubility (less than 1 μM), trans-resveratrol in plasma is extremely poor water-soluble and has a short half-life (8–14 min). It also changes to the less active cis form. Incorporating polyphenols into water-soluble carriers, like liposomes, might provide chemical and biological protection to get around the solubility limitations [26,27]. Several authors investigated exploring liposomes as a potential delivery system for resveratrol in an attempt to overcome the limitations of their low bioavailability and solubility. It was found that the precise position of resveratrol within the liposomal bilayer was crucial in boosting the cell-defence system, preventing resveratrol cytotoxicity, and ensuring the long-term stability of the compound [21,22,27,29].

In selecting the doses for resveratrol and liposomal resveratrol, we relied on established studies. Mahmoud et al. demonstrated the cardioprotective effects of a 20 mg/kg dose in a model of doxorubicin-induced cardiotoxicity, providing a basis for its efficacy [30]. Alhusaini et al. also used this dose to show hepatoprotective effects in a rat model of liver injury, further validating its biological significance [31]. Meanwhile, Ogita et al. explored a higher dose of 50 mg/kg for preventing noise-induced hearing loss, suggesting that increased doses might be necessary for specific conditions [32]. Kodali et al. found that a 40 mg/kg dose effectively mitigated age-related cognitive and mood dysfunctions, which informed our decision to use this dose for liposomal resveratrol to potentially enhance its bioavailability and therapeutic effects [33].

To translate these findings to human applications, we used allometric scaling to estimate the appropriate human doses. The 20 and 40 mg/kg doses in rats correspond to approximately 3.2 mg/kg and 6.4 mg/kg in humans, respectively [34]. For an average human weighing 70 kg, this equates to daily doses of about 224 mg and 448 mg. It is important to note that the absorption, bioavailability, and metabolism of resveratrol in humans are critical factors to consider. The oral absorption of resveratrol is approximately 75% and occurs mainly through transepithelial diffusion; however, extensive metabolism in the intestine and liver results in an oral bioavailability considerably less than 1% [35]. Furthermore, dose escalation and repeated administration of resveratrol do not appear to significantly alter this low bioavailability. This comprehensive review of the literature, along with the considerations of human absorption and metabolism, guided our dosing strategy, ensuring our study contributes valuable insights into the therapeutic applications of resveratrol.

Our goal is to characterize a marketed resveratrol-loaded liposomal formulation for continued preclinical evaluation and to provide preliminary evidence of its stability. The marketed resveratrol-loaded liposomes exhibited average vesicle sizes of less than 200 nm, with a narrow polydispersity index (PDI < 0.250), indicating a uniform size distribution. Additionally, the formulations demonstrated a negative zeta potential of approximately – 25 mV, which is attributed to the inclusion of DSPG as an anionic lipid. This negative charge can enhance the incorporation of resveratrol through ion pairing within the lipid bilayers, thereby improving the overall stability and efficacy of the formulation. Furthermore, the encapsulation efficiency (%EE) exceeded 65%, suggesting that a significant amount of resveratrol is effectively retained within the liposomes. These characteristics are promising for the continued development and application of this formulation in preclinical studies [21,22,29].

Emerging evidence implicates oxidative stress, inflammation, and apoptotic signaling are increased in diabetes onset and progression [36–38]. Hyperglycemia-induced overproduction of reactive oxygen species activates downstream pathways worsening insulin resistance and secretion [39]. It also triggers nuclear transcription factors provoking cytokine release and tissue damage [40,41]. Related signaling cascades further drive apoptosis. Therefore, targeting oxidative stress-inflammation-apoptosis pathways could mitigate diabetes and associated microvascular and macrovascular complications [40,42].

Resveratrol has demonstrated promising anti-diabetic effects in animal and human studies [43–45]. In STZ-diabetic rats, resveratrol administration has been shown to lower blood glucose, improve insulin sensitivity, protect pancreatic beta cells, reduce oxidative stress, and ameliorate diabetic complications. Human studies have also shown improved glycemic control, reduced HbA1c, enhanced insulin sensitivity, and potential protection against diabetic complications. Resveratrol's mechanisms of action involve AMPK/NRF2 activation, insulin signaling modulation, oxidative stress reduction, beta-cell protection, and mitochondrial function enhancement. A major challenge for the therapeutic use of resveratrol is its poor systemic bioavailability after oral administration. Absorption is 70% but rapid first-pass metabolism reduces exposure [46]. Novel formulations like liposomes and nanoparticles are being developed to enhance delivery [18]. Notably, liposomal resveratrol, a formulation that encapsulates resveratrol in liposomes, has been shown to have superior bioavailability compared to conventional resveratrol. Liposomes, being microscopic vesicles composed of phospholipids, can effectively protect resveratrol from degradation and enhance its absorption, leading to improved bioavailability and potentially greater therapeutic efficacy [47]. Considering the efficacy of resveratrol in treating diabetic complications, including nephropathy, retinopathy, and cardiomyopathy, we hypothesized that liposomal resveratrol (LR) could potentially ameliorate diabetes-related cardiac damage in an animal model. Hence, this study aimed to investigate the cardioprotective effects of LR in the STZ-rat model. The blood samples were collected directly from the heart of anesthetized rats. The amount of blood that can be taken from the heart of these animals typically ranges from 8 to 10 milliliters [48]. We investigated LR and resveratrol effects on serum biomarkers related to glycemic control, dyslipidemia, inflammation, oxidative stress, and apoptosis in STZ-diabetic rats. Streptozotocin causes selective beta cell destruction and insulin deficiency, representing a model for type 1 diabetes [49,50]. As expected, streptozotocin-treated rats exhibited marked hyperglycemia indicating uncontrolled diabetes. Resveratrol administration dose-dependently decreased elevated blood glucose levels demonstrating its antihyperglycemic efficacy. The present study further extends these findings by demonstrating the efficacy of LR in modulating serum biochemical markers associated with cardiac complications. At rest, cardiomyocytes maintain a low cytosolic calcium (Ca2+) concentration. However, electrical stimulation depolarizes the cardiomyocyte membrane, activating the slow L-type Ca2 + channels. This activation leads to a minor Ca2 + influx into the cell, subsequently triggering a significant Ca2 + release from the SR into the cytosol [39,51]. LR's observed restoration of total protein levels and calcium homeostasis suggests its ability to protect against diabetes-induced metabolic derangements and cardiovascular dysfunction. The increase in total protein levels in diabetic rats treated with LR signifies a crucial improvement in their health status, as low total protein levels often indicate kidney damage and protein loss through urine (proteinuria), a common complication of diabetes. LR appears to protect and enhance kidney function, reducing protein loss and restoring normal blood protein levels, which in turn reduces cardiovascular risk associated with proteinuria. Additionally, improved protein levels indicate better metabolic health, including enhanced glucose and lipid metabolism, thereby lowering the risk of diabetic cardiomyopathy and other heart-related complications. These findings highlight the potential of LR in mitigating diabetic complications and promoting better heart health in diabetic individuals. The significant reduction in LDH levels following LR treatment further supports its potential to mitigate tissue damage and cellular stress associated with diabetes, as increased LDH levels are commonly linked to conditions like tissue injury, necrosis, hypoxia, haemolysis, and severe inflammation. Elevated LDH activity can disrupt regular glucose metabolism and insulin secretion, attributed to heightened mitochondrial membrane potential, cytosolic free ATP, and cytosolic free Ca2 + in islet beta cells. In light of the observed effects on calcium homeostasis and the potential implications for mitochondrial function and cardiac energetics, further investigations are warranted to elucidate the precise mechanisms underlying LR's protective effects. Future studies focusing on the biochemical evaluation of mitochondrial function will provide valuable insights into the role of LR in restoring physiological energetics in cardiac tissue.

Consistent with previous studies [52,53], our findings demonstrate elevated levels of TNF-α, IL-6, and NF-kB in STZ-induced diabetic rats, reinforcing the link between diabetes and cardiac inflammation. LR was administered to rats to treat diabetic cardiomyopathy and the results were dose-dependent. Diabetic rats receiving the 40 mg/kg dosage exhibited significantly lower expression of TNF-α, IL-6, and NF-κB markers compared to those receiving the 20 mg/kg dosage. In contrast, diabetic rats treated with resveratrol at 40 mg/kg had comparatively higher levels of TNF-α, IL-6, and NF-κB markers in their heart than those treated with LR at either 20 mg/kg or 40 mg/kg. These observations support the efficacy of LR administration, specifically at doses of 20 and 40 mg/kg, in reducing inflammation and ameliorating the prognosis of diabetic cardiac complications when compared to the 40 mg/kg dosage of resveratrol alone.

The intricate relationship between metabolic abnormalities and inflammation in diabetes is further complicated by the involvement of oxidative stress pathways. LR's dual antioxidant and anti-inflammatory properties hold promise in disrupting this cycle and mitigating the multi-organ complications of diabetes, such as nephropathy, neuropathy, retinopathy, and cardiovascular disorders [54]. Our findings support LR's ability to target key intersecting pathways involved in oxidative stress and inflammation. Diabetic rats exhibited increased lipid peroxidation and compromised endogenous antioxidant defenses, indicative of heightened oxidative stress. LR markedly ameliorated these alterations, likely due to its direct free radical scavenging and upregulation of antioxidant enzymes [55]. The current study's findings align with previous research on the cardioprotective effects of resveratrol in diabetic conditions which demonstrated that resveratrol administration significantly improved cardiac function and reduced oxidative stress in STZ-induced diabetic rats.

Several lines of evidence also indicate that enhanced apoptotic signaling accelerates cellular injury in diabetes [39]. Hyperglycemia and excess free fatty acids increase mitochondria-associated pro-apoptotic signaling in endothelial cells, beta cells and neurons [56]. Proinflammatory cytokines provoke apoptosis while death of pancreatic beta cells and endothelial cells propagates metabolic dysfunction and tissue damage [57]. Our study found that diabetic rats receiving only the vehicle exhibited elevated apoptosis in cardiac cells, as evidenced by increased expression of Bax and caspase-3 and reduced expression of Bcl-2. Administration of LR at a dose of 40 mg/kg significantly decreased caspase-3 expression, Bax expression, and increased Bcl2 expression, compared to diabetic rats receiving LR at a dose of 20 mg/kg. Compared to LR treatment at both doses, increased levels of caspase-3 expression, Bax expression, and decreased Bcl2 expression were found in 40 mg/kg resveratrol-treated diabetic animals. The anti-apoptotic effects of LR are likely due to its ability to reduce oxidative stress and inflammation, which are two key drivers of apoptosis [47]. LR has been shown to reduce apoptosis in a variety of cell types, including kidney cells, cardiac cells, and neuronal cells [58,59]. For example, studies in rats and mice have shown that LR significantly reduced apoptosis in the hearts of mice with heart failure, and the brains of rats with Alzheimer's disease, respectively [60,61]. This implies that LR inhibits apoptotic pathways, further contributing to its cytoprotective mechanisms.

Collectively, our findings demonstrate LR's multi-faceted therapeutic potential, encompassing antihyperglycemic, antioxidant, anti-inflammatory, and anti-apoptotic properties in diabetic rats. These beneficial effects were corroborated by histological examination of cardiac tissues, which revealed a remarkable improvement in the mitigation of structural alterations following LR treatment. Diabetic rats treated with vehicle only exhibited distinct histological changes, such as nuclei deformities, interstitial edema, and localized cytoplasmic vacuolization, indicative of diabetes-associated cardiac damage. In contrast, LR administration at dosage of 40 mg/kg body weight effectively reinstated the structural integrity of myocardial nuclei and cardiac myofibrils suggesting a reduction in both cardiac and ventricular hypertrophy.

These findings align with previous research on the cardioprotective effects of resveratrol in diabetic conditions. In a research paper it was reported that resveratrol treatment attenuated myocardial fibrosis and improved cardiac function in diabetic mice [62]. Our findings emphasized the enhanced effectiveness of LR in ameliorating hyperglycemia-induced cardiac damage compared to resveratrol alone, although the latter still demonstrated a more significant effect than the vehicle-treated diabetic animals.

## 5. Conclusion

This preclinical study presents compelling evidence supporting the beneficial effects of LR supplementation in diabetes mellitus. LR treatment effectively countered cardiac dysfunction, oxidative stress, inflammation, apoptosis, and elevated LDH levels in cardiac tissues of streptozotocin-induced diabetic rats, demonstrating its potential to mitigate diabetes-associated metabolic, cardiac, and redox abnormalities. In conclusion, this preclinical investigation provides a robust experimental foundation for LR as a promising pharmaceutical intervention against diabetes and its associated complications.

## Data Availability

The data presented in this study are available upon request from the corresponding author.
